# Solving Man-Induced Large-Scale Conservation Problems: The Spanish Imperial Eagle and Power Lines

**DOI:** 10.1371/journal.pone.0017196

**Published:** 2011-03-02

**Authors:** Pascual López-López, Miguel Ferrer, Agustín Madero, Eva Casado, Michael McGrady

**Affiliations:** 1 Cavanilles Institute of Biodiversity and Evolutionary Biology, Terrestrial Vertebrates Group, University of Valencia, Paterna, Valencia, Spain; 2 Biodiversity Conservation Group, Doñana Biological Station, Spanish National Research Council, Sevilla, Spain; 3 Consejería de Medio Ambiente, Junta de Andalucía, Sevilla, Spain; 4 Fundación Migres, Sevilla, Spain; 5 Natural Research, Ltd, Krems, Austria; Texas A&M University, United States of America

## Abstract

**Background:**

Man-induced mortality of birds caused by electrocution with poorly-designed pylons and power lines has been reported to be an important mortality factor that could become a major cause of population decline of one of the world rarest raptors, the Spanish imperial eagle (*Aquila adalberti*). Consequently it has resulted in an increasing awareness of this problem amongst land managers and the public at large, as well as increased research into the distribution of electrocution events and likely mitigation measures.

**Methodology/Principal Findings:**

We provide information of how mitigation measures implemented on a regional level under the conservation program of the Spanish imperial eagle have resulted in a positive shift of demographic trends in Spain. A 35 years temporal data set (1974–2009) on mortality of Spanish imperial eagle was recorded, including population censuses, and data on electrocution and non-electrocution of birds. Additional information was obtained from 32 radio-tracked young eagles and specific field surveys. Data were divided into two periods, before and after the approval of a regional regulation of power line design in 1990 which established mandatory rules aimed at minimizing or eliminating the negative impacts of power lines facilities on avian populations. Our results show how population size and the average annual percentage of population change have increased between the two periods, whereas the number of electrocuted birds has been reduced in spite of the continuous growing of the wiring network.

**Conclusions:**

Our results demonstrate that solving bird electrocution is an affordable problem if political interest is shown and financial investment is made. The combination of an adequate spatial planning with a sustainable development of human infrastructures will contribute positively to the conservation of the Spanish imperial eagle and may underpin population growth and range expansion, with positive side effects on other endangered species.

## Introduction

Global energy consumption has increased exponentially since the Industrial Revolution, with a concomitant increase in the network of electrical transmission line across the landscape. The development of this network has had effects on animals which are sometimes favorable (e.g. increasing the availability of places for bird nesting on the pylons) and sometimes not (e.g. increased risk of electrocution for some birds, mammals and reptiles and collisions by birds) [1]. Electrocution of birds has been reported to be an important mortality factor [1]. There has been increasing awareness of this problem amongst land managers and the public at large, as well as increased research into the distribution of electrocution events and likely mitigation measures that might be adopted. Mortality caused by electrocution due to poorly-designed pylons and power lines can seriously affect avian species particularly when population size of the affected species is low or its distribution is limited. Under this situation, avian power line mortality becomes a likely cause of population decline, especially when combined with other causes of human-induced mortality such as shooting, poisoning, trapping, collision with human-made objects (e.g. wind turbines), habitat destruction or exposure to environmental contaminants [2]. Potential impact of electrocution and collision with electricity infrastructure is particularly significant for Falconiformes, because their morphology and behaviour make them more prone to electrocution, but Ciconiformes, Strigiformes and Passeriformes may also be affected [3], [4]. Although the problem is clearly identified and some potential solutions have been put forward, land managers still need to convince the public, and especially politicians, about the necessity of adequate investment in conservation programs (including mitigation) aimed at reducing man-induced mortality risks.

Electrocution risks are influenced by many factors that can be divided into two groups: landscape factors and individual factors [2]. The former comprise vegetation structure and composition, landscape topography, prey density and perch availability. Individual factors include pole-top configuration, clearances among electrical components, raptor morphology and raptor behaviour. For example, inexperienced immature and subadult birds [5], [6] as well as females (larger than males in species with reversed size dimorphism such as raptors) are more prone to electrocution than other birds [7], which could result in global population effects. Furthermore, electrocution has been shown to be the main cause of declines in one of Europe's rarest raptor, the Bonelli's eagle (*Aquila fasciata*) [8], [9] and the reduction of electrocution mortality, principally of subadult birds, is likely to be critical to the survival of this endangered species in the Iberian Peninsula [10]. Resolving electrocution problems has been critical to the maintenance of other raptor species such as the endangered Spanish imperial eagle (*Aquila adalberti*) [11], [12] or the Eurasian eagle owl *Bubo bubo* [13], [14] in Europe, or the critically endangered Californian condor (*Gymnogyps californianus*) in North and Central America [15].

Currently, the causes of electrocution in birds have been identified and several measures with different successful results have been undertaken. Fortunately, electrocution risk to birds is not described by a random distribution, but a quasi-Poisson one in which few pylons account for most electrocutions and hence, mitigation is feasible [12]. For example, identification of dangerous pylons and the application of appropriate insulation techniques has resulted in lower electrocution rates whereas other mitigation measures such as perch deterrents have not succeed [1], [16].

The network of above ground power lines has grown continuously in the last 50 years and this has been accompanied by an increase in other man-made structures, including roads, railroads and new power generation facilities (e.g. solar panel fields and wind farms). At least at the local level, once mortality sources have been identified (e.g. electrocution and collision with power lines), their impact can be lessened. However, it is not entirely clear that lessons learned and actions undertaken at the local level can be applied across a wider area to solve large scale conservation problems such as bird electrocution. Here we provide information and a practical example of how mitigation measures implemented on a regional level under the conservation program of the Spanish imperial eagle have resulted in a positive shift of demographic trends of one of the most endangered raptors in the world.

## Materials and Methods

### Study species

The Spanish imperial eagle (*Aquila adalberti*) is a long-lived resident tree-nesting raptor endemic of the Iberian Peninsula [12]. With an estimated population of 250 pairs (National Working Group, unpublished data 2008), it has been considered one of the most endangered raptors in the world [12], [17]. The Spanish imperial eagle population has increased in most of the Iberian populations since 2000. Its main threats are mortality caused by electrocution, poisoning, habitat fragmentation, shooting and the decline of its main prey, the European rabbit (*Oryctolagus cuniculus*) due to viral haemorrhagic disease [18], [19]. Electrocution on power lines has been reported to be the main known cause of death for the species, accounting for 60% of mortality cases [7] with a strong sex-biased distribution towards female birds. This loss of individuals and resultant bias in the sex ratio in the small Doñana population, coupled with the K-species characteristics of the Spanish imperial eagle have been suggested as the cause of the rapid population decline there [12], [20].

### Study area and mortality records

**Figure 1:**
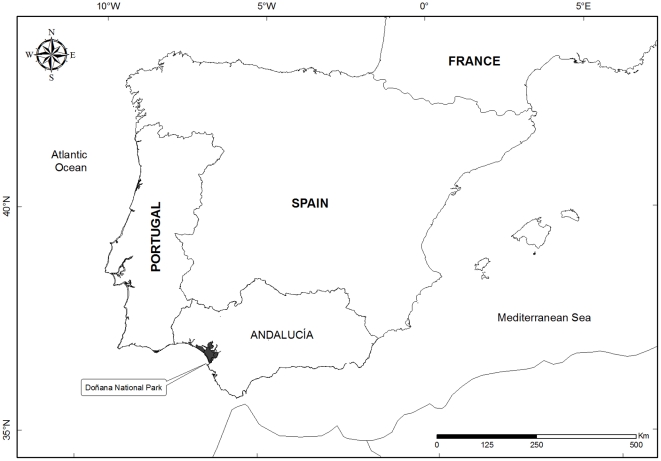
Location of Andalucía and Doñana National Park within the Iberian Peninsula.

The study area included the Andalucía region (area  = 87598 km^2^, southwestern Spain), representing the 17,3% of the total surface of Spain, and the Doñana National and Natural Parks (total area  = 53709 km^2^, 37°N 6°30′W) (Figure 1). A 35 years temporal dataset (1974–2009) on mortality of Spanish imperial eagle was recorded. This included results of population censuses, and data on electrocution and non-electrocution of birds recovered in both the Doñana (local level) and Andalucía populations (regional level). Data from Doñana were obtained from the Doñana field diary (Doñana Biological Station archives) and from the Station Ringing Department. In addition, from 1986 to 1988, data were obtained from 32 young Spanish imperial eagles equipped with solar radio-transmitters (Type HSPB 1400 3xA, Wildlife Materials Inc.), fitted as backpacks to young when they were 50–60 days old [7]. Also, a compilation of data on eagle mortality was obtained from the regional government (Consejería de Medio Ambiente, Junta de Andalucía) which kept records as part of the regional conservation program for this species.

### Mortality surveys

Data on dead birds were gathered by means of specific field surveys during different time periods as follows. Every two months searches of the ground under 100 km of medium-tension power line in and around territories of Spanish imperial eagle were made during 1980–1982 [21], [22]. From 1987 onwards the ground under an additional 500 km of power lines was checked every two months in juvenile dispersal areas as determined by radio-tracking [7]. Furthermore, from 1990 to 1994 a total of 6288 selected power poles were surveyed annually throughout the entire distribution area of Spanish imperial eagle (including both natal and dispersal areas of immature birds), as well as adjacent areas normally beyond the species' range, to assess mortality in other species [23]. Since 1995 until 2009, regular annual surveys of power lines in breeding and dispersal areas have been conducted. During the entire period, we also get additional data from radio tagged individuals (around 150 individuals in total). A detailed description of field methods is available in [7], [22], [23].

Raptor electrocution mainly occurs at low-tension power lines (16–45 kV) [1]. However, in contrast to higher-tension power lines that pertain to a few large companies for which detailed data are available, accurate data about the extension of low-tension power lines were not available for the entire study area given the many owners of particular power lines and the lack of a compiled Geographical Information System (GIS) database. For this reason, data concerning the extension of medium-tension (200–110 kV) distribution power lines were recorded as an adequate surrogate of the low-tension wiring network extension in Spain. These data were obtained from the Spanish Association of Electricity Industry (www.unesa.es) [24].

### Mitigation measures

Mitigation measures included the identification of mortality hotspots, development of predictive cartography and prospective modeling. Both proactive actions (making safe dangerous pylons before mortalities occurred) and reactive measures (making safe after mortalities were recorded) were undertaken. Since pylon design and habitat had highly significant effects on raptor mortality, accounting for 82% of the variance in death rates [22], mitigation measures aimed to ameliorate the impact of power lines were implemented. These included the construction of new pylons with suspended insulators, avoiding the use of pylons with an exposed loop of wire above the insulator, and ensuring that new power lines were located away from both breeding areas and areas of temporary settlement of juveniles eagles [12], [25]. In the case of existing power lines measures were focused on correcting dangerous pylons by replacing exposed rigid insulators with suspended ones and installing protective systems on the pylons to prevent birds from coming into contact with wires [25], [26]. The selection of priorities in retrofit dangerous power poles was made using information on factors affecting mortality distribution [22].

### Statistical analysis

Data were divided into two different periods: before and after the approval of the regulation of power line design in 1990 by the Andalusian regional government (Regulation 194/1990, June 19^th^ of the Junta de Andalucía). This regulation established mandatory rules aimed at minimizing or eliminating the negative impacts of power lines facilities on avian populations in and close to natural protected areas in the region. Although the Regulation was approved in 1990, the first mitigation measures were applied in 1992, so the two periods that were considered in the analysis were 1974–1992 and 1993–2009.

We compared the number of birds found electrocuted per nesting pair and per km of medium-tension power lines at both regional and local levels, between the two periods using a Mann-Whitney non-parametric test [27]. Linear regression curves including each variable versus time were fitted for the two different periods. The slopes of the two curves (before and after) were calculated and the change of the value assessed. Homogeneity of slopes tests were used to examine the interaction between the period and each of the continuous variables in influencing responses [27]. The significance threshold was set at alpha  = 0.05. Statistical calculations were performed within STATISTICA 7.0 [28].

## Results

**Figure 2:**
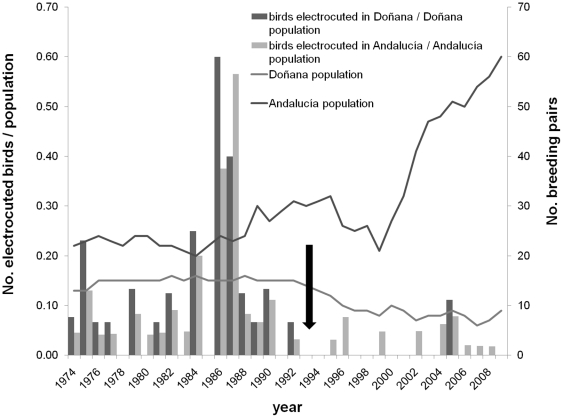
Annual recorded electrocution mortality of Spanish Imperial eagles. Data are shown as a percentage of population in Doñana and Andalucía in relation to population trends in the two areas. The black arrow shows when mitigation measures were implemented in Andalucía (Regulation 194/1990, June 19^th^ of the Junta de Andalucía).

Since 1974, a total of 158 Spanish imperial eagles have been recorded dead in Andalucía, 101 of them (63.92%) inside Doñana National Park. Electrocution was the most frequent cause of death accounting for 39.87% of the total mortality events. Since 1974 when the first death by electrocution was recorded, 37 Spanish imperial eagles have been found electrocuted in Doñana (36.63% of the mortality cases) and 26 cases in Andalucía (41.94% of the records) (Figure 2).

The population of Spanish imperial eagle has increased in Andalusia from 22 pairs recorded in early 70s to 60 pairs recorded in 2009 (average annual percentage of population change  = +3.46%), with this population currently representing 24% of the world population of the species. However, the Doñana's population, which is separated from other breeding populations (the nearest nesting conspecifics breed 300 km away; [12]), has not experienced a parallel increase during the same time period, starting from 13 pairs in the 70s, reaching up to 16 pairs in the 80's and followed by a period of decrease in the 90's and early 2000 (average annual percentage of population change  = −0.40%). In relation to the two time periods considered, before and after the approval of the Andalusian regulation of power line design, the average annual percentage of population change has increased from +2.24% to +4.74% for the Andalucía population and decreased from +0.92% to −1.80% in the Doñana population. In the latter case, after 1992, a dramatic increase in annual adult mortality due to an increase in illegal use of poison in hunting areas surrounding Doñana National Park was recorded. Currently, the Doñana population is increasing slightly; nine pairs bred in 2009 (Figure 2).

**Figure 3:**
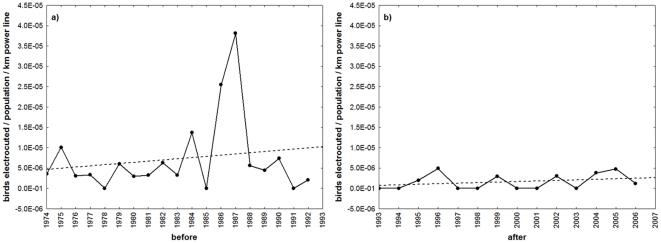
Numbers of Spanish imperial eagles electrocuted in Andalucía (Southern Spain). Data are shown as the number of birds electrocuted per nesting pair and per km of medium-tension power lines (200–110 kV): A) before, and B) after the implementation of mitigation measures included in the Andalusian regulation of power line design. Regression (broken) lines are shown.

In relation to mortality causes, the number of birds recovered dead by any cause has decreased considerably in Doñana (−72.40% of change in the cases recorded before and after the mandatory regulation was approved in 1990) and Andalucía (−45.17% reduction). Comparing the same time periods, both Doñana (−96.90%) and Andalucía (−61.95%) experienced declines in the number of dead eagles being recorded as victims of electrocution. Significant changes in the slope of regression curves between the two periods are reported in Table 1. Although mortality between these two periods decreased there was a continuous increase in the amount of overhead electrical wiring at regional and national levels. Interestingly, data show a significant change in the number of birds found electrocuted per nesting pair and per km of medium-tension power lines both in Andalucía (Mann-Whitney, U = 58.50, Z = 2.75, P = 0.006) and in Doñana (Mann-Whitney, U = 43.50, Z = 3.56, P<0.001) (Figure 3). Since 1992 until 2009 a total of 6560 dangerous pylons were made safe along 1446 km of power lines in Andalucía. The total budget for these measures amounted €2,624,000 (an average of €400.00 per corrected pole). This figure represents slightly over half of the total investment in conservation of the species for this period which adds up a total of €4,481,665.12.

**Table 1:** Comparison of variables before (1974–1992) and after (1993–2009) the approval of the Andalusian regulation, which established mandatory rules to minimize and eliminate the negative impacts of power line infrastructure on avian populations.

Variable	*F*	*p*	% change in slope
No. of birds electrocuted in Doñana	8.589	0.006	−84.494
No. of birds electrocuted in Andalucía	2.551	0.120	−48.998
extension of medium-tension wiring (200–110 kV)	53.415	<0.001	−29.612
electrocution in Doñana * Doñana population^−1^* extension of the medium-tension wiring (200–110 kV)^−1^	8.610	0.006	−6.023
electrocution in Andalucía * Andalucía population^−1^* extension of the medium-tension wiring (200–110 kV)	4.810	0.036	−53.939

## Discussion

Large scale conservation problems and consequent appropriate management actions operate at different temporal scales. Whereas human-induced factors affecting mortality may operate in relatively short time frames (e.g. bird electrocution, wind farm collisions, shooting, poisoning, etc.), management actions require longer temporal scales in order to determine how effective really are. This leads to conflicts between politicians, managers of the public administrations and scientists, whose relevant timeframes differ from one another. In the situation we describe, long-term data series of both population and mortality for Spanish imperial eagle are essential in determining the effectiveness of mitigation measures and their likely effect on population persistence. Therefore, we used a 35-years data series of the Spanish imperial eagles' population from Doñana and Andalucía to report a well-studied example of how a large scale conservation problem has been identified and how, after applying evidence-based mitigation measures, there has been a positive shift in the demographic trend of one of the most endangered raptors of the world.

Our results show a shift in the main causes of mortality between the two periods, before and after the approval of mandatory regulation against bird electrocution in the Andalucía region. Whereas during 1973–1992 the main problem was electrocution [7], after 1992 the main mortality source shifted to the illegal use of poison in the Doñana surrounding area [29]. In late 80's and early 90's, several studies highlighted the risk of electrocution to population persistence of the Spanish imperial eagle [7], [21], and mitigation measures were instigated accordingly. As a consequence, electrocution rates have changed from accounting for nearly 60% of total mortality events [7], to 39.87% (this study). In a general review of raptor electrocution studies, Lehman et al. [2] stated that “without information on bird numbers, a post-mitigation decrease in mortality could be attributed to pole modifications, when in fact it is the result of a population decline”. This is not the case of Spanish imperial eagles for which the reduction in electrocution fatalities has been accompanied by a general increase in the population, and demonstrates that at least some large scale conservation problems can be resolved. However, the question remains as to the total cost of fixing the problem, and whether that cost is affordable.

In seeking affordability of mitigation methods it is important to try to maximize benefit while minimizing cost. One of our main recommendations in solving an electrocution problem is to concentrate on the most dangerous pole designs. In our study, there was an association between pole design and habitat type. For example, pin-type poles in the natural habitat accounted for 26.3% of the mortality of Spanish imperial eagles [23], but only 6.7% of poles in natural habitat are of this type. So, mitigation measures aimed at correcting pin-type poles not only achieved the aim of reducing mortality, but were cost effective. Our results show how, after mitigation, there has been a strong decrease in bird electrocutions, both in the Doñana and the Andalusian populations, with a 97% and 62% of reduction respectively. This is clearly good news from the conservation point of view and well exceed calculations of Janss and Ferrer [23], who forecasted a 79.7% reduction in mortality as a result of correcting 37.2% poles in Doñana.

The importance of human-induced mortality should also be considered in the context of population dynamics. Demographic variables are not independent of one another when endangered small populations are analyzed, as the case of Spanish imperial eagles [29], [30]. In long-lived spatially structured populations, pre-adult and adult mortality play a key role in influencing population persistence [31]–[33]. Therefore conservation measures should be focused on mitigating detrimental effects on these parameters [10], [30]. In the case of Spanish imperial eagles, adult survival is higher than pre-adult and floater survival and hence small electrocution rate of adult eagles leads to more severe consequences on population dynamics than the death of non-breeding individuals [34].

During the period 1995–2001 several projects carried out by the Autonomous Communities (including Andalucía) and the Spanish Ministry of the Environment were implemented to protect and conserve the Spanish imperial eagle, and some of these included measures to avoid bird electrocution. In addition, there has been an increasing pressure to legally require that efforts to mitigate avian electrocution are made. As a consequence, regional and national laws were approved with regard to avian electrocution (Decree 178/2006, October 10^th^, of the Junta de Andalucía), “that establishes mandatory rules for the protection of avifauna against high-tension power lines”, and the Spanish Royal-decree (263/2008, February 22^nd^), “which establishes mitigation measures in high-tension power lines to protect birds”. Mitigation measures included the insulation of cross arm braces, which was the most effective and practical tool to reduce electrocution of raptors at metal lattice power poles [35], taking into account that insulating materials should be periodically checked and replaced. Other effective measures included the fixation of spirals and the crossed bands for conductor-marking and the installation of static wire-marking to avoid collisions [36]. In contrast, other actions such the employment of raptor models as deterrents were not effective [16].

### Conclusions

Conservation and the preservation of biodiversity require financial investment from both the public and private sector budget. In our case, nearly €2.6 million have been spent on mitigation of bird electrocution during 1992–2009, which equals an investment of €154,352.94 per year. The Spanish imperial eagle population in Andalucía has increased from 31 to 60 pairs in the same period. Taking into account the high budgets assigned to the construction of new power lines and alternative power sources (e.g. wind farms, solar panel arrays), our results demonstrate that solving bird electrocution is an affordable problem if political interest is shown and financial investment is made. Furthermore, other avian species could have benefited from these measures [2], [10].

Future research should be focused on the development of new high-efficiency, low-cost devices that reduce electrocution risk of distribution power lines. These mechanisms should be easy to install. They should allow birds to perch and nest while protecting the lines to ensure that power supply is not disrupted due to bird electrocution events or short-circuiting by nest material. Finally, conservation actions should include adequate spatial planning, avoiding the placement of power lines in areas of special conservation interest. The combination of an adequate spatial planning with a sustainable development of human infrastructures will contribute positively to the conservation of the Spanish imperial eagle and may underpin population growth and range expansion, with positive side effects on other endangered species.
